# Impacts of Hospital Acquired Bloodstream Infections in Patients Undergoing Hemodialysis Through a Central Venous Catheter

**DOI:** 10.1017/ash.2024.200

**Published:** 2024-09-16

**Authors:** Mary Michaud, Sharada Weir, Peter Sullivan, Elizabeth Hurlburt, Jared Crandon

**Affiliations:** Cormedix Inc.

## Abstract

**Background:** Hospital acquired infections (HAI) are of interest given their resultant morbidity, mortality, and hospital utilization. Among HAIs, central line associated bloodstream infections result in the highest rates of mortality and additional costs. While all central venous catheters (CVC) carry risk for BSI, long-term catheter use is at increased risk. One population that utilize CVCs for extended durations are those undergoing hemodialysis. While data are available characterizing BSI impacts on outpatient hemodialysis patients, little data exist describing inpatients. The purpose of this study was to characterize the demographics, outcomes, and economics associated with the development of hospital acquired BSI (HA-BSI) in patients undergoing hemodialysis through a CVC (HD-CVC). **Methods:** All admissions of adult patients in the Premier Healthcare Database with hospital stays including HD-CVC with discharge dates during 2020-2022 were retrospectively evaluated. BSIs were identified by ICD-10 codes and blood culture collection dates. A BSI was deemed hospital acquired if the blood culture date was ≥3 calendar days after admission. Descriptive analyses were undertaken for HA-BSI patients including: baseline demographics, clinical characteristics, and outcomes. Length of stay (LOS), ICU utilization, and estimated costs were evaluated for HAI-BSI and non-BSI populations. **Results:** 166,394 admissions from 91,448 patients were identified. Of these, 5,722 patients (6.3%) had 5,842 admissions with a HA-BSI. These patients were 58.9% white, 28.3% black, 56.8% male, and 62.9% were aged ≥60years. Patients had considerable comorbidities at baseline with 88.9% having ≥2 Charlson comorbid conditions and 46.9% with ≥6. During the study period, all-cause mortality was 27.8% for HA-BSI patients with 85.5% of deaths occurring while inpatient. Median LOS for patients with HA-BSI was 25 days compared with 6 days for HD-CVC without BSI; patients with HA-BSI were also more likely to require the ICU (65.6% vs. 27.6%). The median ICU LOS was 12 days for HA-BSI versus 34 days for HD-CVC without BSI. Greater intensity of healthcare utilization was reflected in median costs of $402K for HA-BSI, compared with $43K for HD-CVC without BSI. Discussion: We described the characteristics of HD-CVC patients that developed HA-BSI. These patients had many comorbidities and relatively high rates of all-cause in-hospital mortality. Patients were likely to have long LOS, both in-hospital and within the ICU. Collectively, care of these patients was associated with considerable healthcare costs, particularly as compared with HD-CVC patients not developing a HA-BSI. Future studies should characterize risk factors and evaluate potential prevention strategies for this high-risk population.

